# Is mobile app a new political discussion platform? An empirical study of the effect of WeChat use on college students’ political discussion and political efficacy

**DOI:** 10.1371/journal.pone.0202244

**Published:** 2018-08-09

**Authors:** Hua Pang

**Affiliations:** Institute of Media and Communication, Dresden University of Technology, Dresden, Saxony, Germany; Institute for Complex Systems, CNR, ITALY

## Abstract

In the last couple of years, the increasing application and popularization of mobile app have dramatically transformed people’s daily political lives through offering innovative mechanisms for interpersonal communication. While a majority of past studies on WeChat have mainly focused on its characteristics, only few documents have unearthed the potential effect of using such emerging social media on facilitating political discussion and increasing political efficacy. Given those, this study adopted uses and gratifications approach to explore the possible relationships between gratifications-sought, the intensity of WeChat usage on mobile phones, online political discussion, and political efficacy among college students in China. An empirical survey with 282 WeChat users reveals that WeChat as a relatively new outlet of political information, which fosters online political discussion with others about government and politics. Contrary to the expectation, the intensity of WeChat usages is not helped to strengthen or enhance individual’s level of internal or external political efficacy. Furthermore, hierarchical regression analyses demonstrate that information needs and recognition needs are positive predictors of internal political efficacy. Overall, these obtained findings may shed more up-to-date insights on the meaningful role of the mobile-based communication technology in promoting citizens’ democratic engagement in a developing country.

## Introduction

In today’s China, the dramatic development of mobile phone platforms and widely popularization of wireless communication technologies make the birth of the mobile Internet era. By the end of December 2017, the overall population of Chinese ‘mobile netizens’, who connect to the network via mobile devices, increased to approximately 753 million, accounting for 97.5 percent of the total number of the Internet population in China based on statistical data from China Internet Network Information Center [1]. Meanwhile, as smartphone and mobile web usage continue to soar, WeChat as the most extensively utilized mobile social networking application has drawn considerable attention among mobile web users [2]. Presently, with a total of roughly 693 million active users, the tremendous popularity of WeChat has made itself become a major online source of news and private communication channel for Chinese people [3].

Owned by Chinese company tech giant Tencent, WeChat (literally: “micro message”) was released in China on January 21, 2011 [4]. It is not only a domestic instant messaging mobile service that allows users to send voice, video, pictures or text information covering a wide variety of subjects from individual interests and preferences to current political events in real-time, but also a hybrid technology that combines a series of services including group chatting, mobile games, online payments and so on [5]. Moreover, the innovative mobile application is completely free to install, use, download, and supports almost every mobile phone devices consisting of iPhone system, Android system, BlackBerry system as well as Windows Phone operating system [6].

Thanks to its converged technological features, this newly technology provides a more information-rich space where people can conveniently seek and distribute information online, browse the latest political coverage and international breaking news, sustain interpersonal relationships, as well as exchange personal opinions through their interpersonal network ties [7–9]. Additionally, with the economic development in recent years, Chinese citizens are more likely to engage in diverse political activities due to they have more political knowledge as well as enthusiasm for the political process than before [10]. However, Chinese government’s tight control of the Internet since late 2008 has led to thousands of web sites, both foreign and domestic, were blocked for touching on some politically sensitive topics [11, 12]. Under such circumstance, relatively more freeway has been given to WeChat through mobile devices.

Yet, despite WeChat has seamlessly infiltrated every aspect of Chinese people’s life and helped generate innovative means of connection and communications, there is seldom empirical research of the powerful impact of it on individual’s civic and political life. Taking consideration of WeChat has attracted a large number of younger generation, around 76 percent of its users in China are between 22 and 30 years old [13], this study thus begs these overarching questions of: whether WeChat has become a crucial political discussion ground in Mainland China among the college student population? Furthermore, to what extent is WeChat use associated with individual’s external and internal political efficacy? To address these concerns, the present study examines the influence of WeChat in politics by looking specifically at Chinese WeChat users combined the theory of uses and gratifications. The results of the article may allow media researchers and practitioners to obtain a more sophisticated understanding of the underlying mechanisms behind these different linkages and contribute to broader theoretical studies on the actual impact of the new digital technology on democracy development in contemporary China.

## Literature review

### Understanding social media use through mobile devices

Along with the widespread and significant of new digital media in the political life of people, the impact of social networking sites through mobile phones on Chinese politics is gradually becoming a hot topic in contemporary academic debate. Although scholars deem that continuous penetration of the new media technology brings about additional issues to the fore and challenges to the traditional state power [14], this has little systematic research results in an authoritarian regime, not to mention any conclusion. As Chen claimed that, studies on how social media affect political mobilization in China are still extremely scarce, especially for relevant theoretical work and empirical researches [15]. Actually, the ongoing debate remains between cyber-realists and utopians regarding the uses and effects of social media [16, 17]. Some optimistic scholars suggested that the adoption of social media will stimulate political activism and social change, which eventually lead to a more pluralistic and democratic China [4, 18]. Nevertheless, some pessimist argued that social media will not bring about such change owed to the ruling Communist Party’s strict Internet censorship [19]. In general, in this debate, social media has consistently been recognized as a mere platform for information [20]; the practical effect of it has been marginalized. Specific speaking, previous research on social media has largely taken two forms: adopting the content analysis method on specific political issues on microblogs [21, 22], or only teleological debates which argue about the implications of social media for substantive political outcomes [4, 16].

The nature of WeChat as one brand new type of social media is known mostly for its private chatting and sharing of photographs and videos among members’ small circles of friends via mobile phone. More specifically, WeChat aims primarily at a small but significant number of friends that users can conveniently communicate and interact with them in everyday life. In addition, WeChat establishes circles of close interactions for sharing useful information easily and instantly, so that users are able to control the flow of diverse information [16, 23]. Compared to microblog, WeChat is more like a personal network. So when some globally popular social media networking sites such as Facebook, YouTube, and Twitter are banned in China, WeChat has gradually become the dominant platform for online political discussions [7]. This indicates that WeChat boosts a hybrid virtual discursive avenue where Chinese citizens, especially college students and young people, can voice and exchange viewpoints on political events and current issues and its decentralized construction can create a new challenge to the government authority through circumventing centralized content control and Internet censorship [3, 24].

Given that millions of Chinese citizens have WeChat accounts in a few years, little research has paid attention to the unprecedented growth of WeChat. Meanwhile, comparison of microblog and other types of social media, WeChat has not been extensively explored in the political communication arena. Until now, only few quantitative researches have thoroughly examined the effect of WeChat use on political behaviors and attitudes among Chinese citizens [11, 15, 24, 25]. For example, Wei and his coworkers suggested that the vast numbers of posts on WeChat’s public accounts would give rise to mobile public spaces for ordinary citizens to participate in civic and political communication [25]. It has thus consequently marginalized questions of WeChat use and the political role of the emerging media. In the case of China, where the state devotes particular effort to constantly surveillance and control of the mass media and Internet, and where a distinctive mobile app has developed, this is an especially serious omission. Hence, the following research questions are advanced: How do individuals engage in political discussion through WeChat in China? How is the intensity of WeChat usage associated with political discussion as well as political efficacy?

### WeChat and online political discussion

With more Internet users engaged in online discussions, a growing number of researchers have argued that there exists a linear relationship between social media use and online political discussion. Due to mass media are strictly controlled by the government in China, the Internet has increasingly become a perfect public sphere for like-minded users to touch on some of politically touchy topics [10]. Previous research has found that communicating about political topics in the computer-mediated setting complements face-to-face political discussion [26]. Indeed, online political discussion through WeChat is available for individuals to express dissatisfaction with governmental authorities and talk about the current political situation, which thereby facilitates a sense of liberalization in cyberspace [3]. Consequently, Chinese citizens can establish a more active and significant association with governments and official institutions as they have more free opportunities to formally influence the government public opinion on criticism of the government and backlash against social injustice. As some scholars elaborated, media use informs individuals about politics; well-informed individuals freely talk about politics, form political opinions, and subsequently participate in the political process [27]. That is, web-based political discussion is as a mediator of media use’s effect on civic and political behaviors. When compared with traditional sources of media, WeChat has the advantages of convenience, lower cost, as well as freedom of expression. Thus, WeChat users in China are not concerned or worried about to be punished by authorities for expressing dissent and criticism. In practical terms, this may imply that the average person could acquire more chances and incentives to involve in political discourse and participate in public affairs. Besides, more and more Internet users seem to form a tacit understanding when discussing taboo topics and can even disseminate the unsanctioned news by creatively using code words with hidden meanings. Given that WeChat as a more private communication platform, people may tend to express their distinctive political opinions online more openly and freely through the media avenue in contemporary China. Thus, the study proposes that:

H1a: Intensity of WeChat usage is positively related to online political discussion about government and political affairs.

### WeChat and political efficacy

The concept of political efficacy originally refers to the belief that individual’s political behavior could exert an influence on the political process [28]. Among the multitudinous factors impacting political behaviors, political efficacy is considered to be one of the most prominent psychological definitions closely associated with individuals’ political actions [29]. Consistent with its key theoretical status, WeChat may increase levels of citizens’ political efficacy because they are able to obtain confidence that they can comprehend the present political issues and join in political discussions if they would like to; and notice that the authority is striving for being accountable to the people and working to benefit them.

Political efficacy includes two distinct dimensions: internal and external efficacy. Among them, internal political efficacy describes an individual’s perception of political competence to know and to engage effectively in a political process. Comparatively, external efficacy refers to citizens’ perceptions that officials and government institutions are likely to be responsive to their needs and interests [30, 31]. In other words, external efficacy represents a feeling that one’s attitude towards the incumbent political regime in general. For instance, people with higher external efficacy tend to believe that their needs will be taken into account by the governmental institutions, and they will therefore deem political engagement to be more effective. Consequently, they may have a higher propensity for political action [32]. Conversely, low external efficacy generally implies apathy toward politics or government, and citizens with a sense that the governmental authorities and institutions do not represent their opinions [33]. Thus, internal efficacy manifests confidence in one’s ability to involve in politics, whereas external efficacy reflects confidence in system responsiveness.

Empirically, previous scholars have applied the definition of internal political efficacy to demonstrate that online media use is associated with increased internal political efficacy in a battery of researches on Internet [34], social media [35], online news [36], and mobile phone [37]. In the context of WeChat use, such a relationship can be expected since information from the new media is a crucial source that can reinforce one’s ability and confidence to engage in politics. Additionally, WeChat may lower barriers to political engagement and improve individual’s internal efficacy by offering an alternative way to interact with activist groups and administration officials.

In terms of citizens’ external political efficacy and their media use, it is relatively small by contrast with internal political efficacy studies. It’s possible because some scholars have primarily concentrated on individuals’ self-perceptions of their own capabilities to participate in political and civic events rather than their attitudes toward the current situation of politics [36]. Furthermore, several scholars are still working on external political efficacy have obtained inconsistent outcomes of the association between media use and external political efficacy. For instance, Kenski and Stroud reported that the Internet may help improve citizens’ external efficacy by enabling them to interact with public officials and other citizens in a virtual network space [38]. However, Chan and his colleagues discovered that intensity of microblog use was negative related to the perception that one’s sense of the government is able to reply to requirements of the regular people [24]. Considering the reality of present China, the Chinese government authorities imposed new Internet censorship measures on the country’s most welcomed mobile instant messaging platforms, those tighter regulations meant to prohibit the posting or reposting of the unauthorized political information and current affairs [39]. For WeChat users, the government’s performances may exacerbate the tension between it and them, leading citizens to doubt that the motives and responsiveness of the government officials. Therefore, the following two hypotheses are formulated:

H1b: Intensity of WeChat usage is positively related to internal political efficacy.
H1c: Intensity of WeChat usage is negatively related to external political efficacy.

### Uses and gratifications of WeChat use

Uses and gratifications (U&G) is a theoretical framework from the field of mass communications research that is utilized to concentrate on individual usage and selection of media [40]. As mentioned previously, the main contribution of this theory is that it premises users are active in the choices of these specialized medium to access certain information; that such options are made based on the perceived psychological needs that are able to be received from the content [40]. With the rapid popularization and development of social media, the U&G perspective is successively applied to individuals’ psychological motivations of using a variety of social networking sites, including microblog, Facebook and Twitter [24, 41]. Recently, Lien and Cao put forward that entertainment, sociality, and information are significant motivations of utilizing WeChat and they are the determinants of Chinese users’ attitudes [6].

In these motivations, information and entertainment uses are identified as two fundamental needs that are widely used to determine the use of virtual communities in the previous political communication literature. Specifically, information need refers to using media to acquire the necessary information that individuals are interested in [2]. It reflects how individuals choose to use media to understand events that are happening and obtain useful information. Entertainment need indicates that using media to have fun, get amusement, and pass the time [2]. Existing studies have demonstrated a stronger link between informational use and political attitudes and behaviors than recreational use. For instance, Park, Kee and Valenzuela revealed that users who seek and find information are more likely to involve in various political activities. Instead, recreational demand is not a vital factor to interpret those political behaviors [42]. Consisted with this outcome, a latest research on smartphone further indicated that informational use of it is significantly associated with political efficacy and participatory behaviors. However, recreational use is not found a positive association with political efficacy [37].

These different results might be explained by the fact that individuals who use the new media for informational purposes probably obtain more mobilizing information and experience more opportunities for engagement in distinct political activities [43]. Furthermore, as people’s general political knowledge increases via social network sites, it may facilitate their media reflection and elaboration, thereby cultivating the better informed citizens, as well as promoting the sense of political efficacy and political engagement [44]. In contrast, students who mainly utilize social media for recreational reasons tend to mostly be involved in the more comfortable and enjoyable modes of activities or organizations, including sport clubs or music groups [42]. As a result, they would not have as strong an emotional attachment to the political causes as to civic actions, such as petitioning, demonstrations, and political talks. Therefore, the entertainment uses of social media might not boost users’ civic engagement and participation behaviors.

Moreover, taking into account the WeChat as a popular mobile app, based on the most relevant findings from the study of motivations of social media use on mobile phone [7, 12] and motivations of online content generation [45], the present study attempts to systematically uncover the gratification factors of WeChat use as well as the relationship between WeChat use and political efficacy among college students. Due to the importance of information and recreational requirements, past research has affirmed the significance of the information and entertainment needs of media use in relation to citizens’ levels of political attitudes and behaviors [24, 45]. According to Tolbert and McNeal, as people became more knowledgeable about political events by browsing recent news reports and information on social media, they were more likely to engage in this issue [46]. Likewise, college students who have more information need are able to decide to use WeChat to discussion political issues with others and feel that they are powerful enough to affect the current politics, and that the governmental authorities are accountable to its citizens. Therefore, it seems reasonable that, informational uses of WeChat will be positively associated with some politically relevant variables, such as political efficacy, political discussion, and political participation in the online political communication area. Conversely, such cognitions could be lower for those who use WeChat primary for recreation and amusement purposes, such as following the fun, playing online games and watching films. Because WeChat users may be distracted by diverse entertainment activities and even pay less attention to the information about political issues and current affairs [3]. Based on the above discussion, the following question is proposed to contribute to a more accurate understanding of motivations for the use of WeChat: How these different dimensions of motivations associate with intensity of WeChat use? In order to answer these questions, it is expected that:

H2a: Motivations of information in WeChat usage is positively associated with discussion about government and politics.
H2b: Motivations of recreation in WeChat usage is negatively associated with discussion about government and politics.
H3a: Motivations of information in WeChat usage is positively associated with internal efficacy.
H3b: Motivations of recreation in WeChat usage is negatively associated with internal efficacy.
H4a: Motivations of information in WeChat usage is positively associated with external efficacy.
H4b: Motivations of recreation in WeChat usage is negatively associated with external efficacy.

## Method

### Participants and procedure

The data utilized in this investigation were based on an online survey. Sampling procedures started with posting links to the online questionnaire on popular forums and social networking sites among Chinese students for two weeks. Subsequently, a total of 282 respondents aged 18 years or older from China filled in the questionnaires from August to September 2015 on www.sojump.com. The questionnaire was designed in Chinese language and all the respondents were selected by using snowball sampling method. Given this sampling strategy is free and enable scholars to target and investigate this specific online population playing around with WeChat, the current sample could be regarded as suitable for the objectives of the research. All respondents were informed about the general purpose of the study in advance and assured that their answers are voluntary and anonymous. The summary of the main data about WeChat use is represented in Tables 1 and 2.

**Table 1:** Summary statistics for WeChat use intensity items (n = 282).

Summary Statistics for WeChat Use Intensity Items	M	SD
About how many friends or acquaintances do you have on WeChat?	1.27	0.6
How often do you use WeChat in a day? (1–4)ª	2.38	1.2
How much time do you use WeChat in a day?	2.58	0.99
How long have registered your WeChat account?	4.07	1.32
How do you agree the following statements?		
WeChat is part of my everyday activity;	3.36	1.03
I am proud to tell people I'm on WeChat;	2.68	0.98
WeChat has become part of my daily routine;	3.29	1.05
I feel out of touch when I haven't logged onto WeChat for a while;	2.45	1.03
I feel I am part of the WeChat community;	2.88	1.06
I would be sorry if WeChat shut down.	2.98	1.09

ªMeans and standard deviations are based on prestandardized figures. They were subsequently recorded to a 1 to 5 range before taking an average to create the scale.

**Table 2:** Principal components factor analysis for WeChat motives (n = 282).

			Factors					
Gratification items	M	SD	1	2	3	4	5	6
**Recognition needs**	**2.96**	**0.89**						
To build up my confidence.	2.86	.913	.824					
To gain respect and support	2.85	.943	.796					
To establish personal identity.	3.05	.944	.665					
Because it is satisfying.	3.07	.953	.658					
To enhance sense of belonging by creating or joining groups.	2.96	.950	.557					
**Fashion or status**	**2.68**	**0.90**						
To look cool	2.68	.911		.883				
To look stylish	2.59	.939		.876				
To look fashion	2.75	.999		.818				
**Affection**	**3.30**	**0.87**						
To get the feeling that people care about me	3.24	.956			.765			
To share position, opinion and personal values	3.41	.968			.678	.407		
To let other know I care for them	3.04	.936			.676			
To share topics with friends	3.52	.873			.640			
**Accessibility**	**3.38**	**0.83**						
To response to friends’ messages	3.65	.805				.728		
To add new friends anytime and anywhere	3.36	.952				.702		
To response to strangers’ requests	2.93	.965		.470		.671		
To be available to friends anytime and anywhere	3.57	.901			.425	.615		
**Cognitive need**	**3.26**	**0.92**						
To find out what is going on in society	3.32	.937					.852	
To understand events happening	3.35	.988					.813	
To broad my knowledge base	3.10	.958	.468				.595	
**Entertainment**	**3.40**	**0.82**						
To pass time	3.43	.906						.805
Because I am curious.	3.29	.939						.723
To have fun	3.49	.863						.611
**Eigenvalue**			11.02	1.90	1.56	1.05	0.85	0.81
**Cronbach’s alpha**			0.90	0.94	0.90	0.85	0.88	0.76
**Variance explained in rotated solution (%)**			14.08	13.81	12.51	10.90	10.42	8.77

Measure scales for gratifications-sought items were from 1 = strongly disagree to 5 = strongly agree.

## Measures

### Dependent variables

#### Online political discussion

It was assessed with a single item which asked the respondents how often they participate in online discussions about the political issues. Participants answered the frequency of discussion according to a five-point Likert-type rating scale ranging from (1) never to (5) daily (M = 2.01, SD = 0.85).

#### Political efficacy

Grounded on a previous study on the effects of Internet use on political variables from questions from Kenski and Stroud, the research adopted the same items to assess political efficacy [38]. All the respondents were asked two questions to gauge these two different dimensions of political efficacy. Respondents were replied according to a 5-point Likert research scale (1 = strongly disagree to 5 = strongly agree). External efficacy was measured by asking participants how do they agree or disagree with the view that people like me don’t have any say about the government authorities do (M = 2.18, SD = 0.94) [38]. Whereas internal efficacy was metered with the statement by asking that how do they agree or disagree that sometimes politics and government appear so intricate that a person like me can’t really know what the hell is happening (M = 3.04, SD = 0.98) [38].

### Independent variables

#### The intensity of WeChat use

WeChat use on mobile phones was evaluated in several items: (1) the frequency of use in a day; (2) amount of time spent on WeChat per day; (3) The history of use; (4) the total number of their friends on WeChat. Besides these four dimensions, the measure of the intensity of WeChat usage also includes a series of attitudinal questions adopted from Ellison et al. [41]. Specifically, this emotional measurement consists of a set of six attitudinal statements to tap the degree to which the participant felt an emotional attachment to WeChat and the extent to which WeChat was woven into their everyday lives. Based on the 5-point Likert rating scale, participants were asked the extent to which they agreed or disagreed with the six statements such as whether WeChat has become a part of their daily activity. Responses to the entire set of nine questions were subsequently averaged to establish a WeChat intensity scale for later study (see Table 1).

#### Motivations of WeChat use

Respondents were given a list of gratification items for the WeChat use adapted from the prior gratification-related literature on the user-generated online content on the Internet and social network site [45, 47]. Because some items were utilized to explore gratification of the Internet and social media use, the study modified these items so that they could better suit WeChat users’ needs. Moreover, all 22 items were replied according to a 5-point Likert rating scale ranging from (1 = strongly disagree) to (5 = strongly agree). They were required to indicate that why they choose to apply WeChat, specific reasons mainly consisting of getting information, seeking amusement, socializing with their own friends and family, and making themselves look fashion (see Table 2).

#### Political interest

Political interest is deemed as a control variable due to it is a crucial predictor of individual’s political act. All students were invited to indicate their level of agreement: how interested are they in government and political events? (M = 3.35, SD = 0.89). They replied on the basis of a 5-point Likert scale varying from (1 = very uninterested) to (5 = very interested).

### Control variables

In the survey, personal demographic information is controlled in the analyses by asking questions mainly including gender, age, education, and annual household income.

## Statistical analysis

Two steps were taken in order to answer the research questions and test the hypotheses. First, principal-components analysis with varimax rotation was run to extract and interpret possible WeChat motive factors. After that, hierarchical multiple OLS regression analysis was implemented to explore the extent to which respondents used the WeChat for online political discussion and political efficacy. Specifically, the variables were entered in different blocks: demographics in block 1, political interest in block 2, WeChat use motivations in block 3, and intensity of WeChat use in block 4. Such a sequential approach not only lets the study evaluate the potential influence of every block of variables on political behaviors, but also enables it to detect the effects of social media attributes controlling one another [48]. All these researches and studies were conducted with the aid of the software SPSS version 22. Online political discussion, internal efficacy and external efficacy were the criterion variables in each of the regression equations.

## Conclusion

### WeChat uses and gratifications

By using the 22 gratification questions generated from the previous literature, the study adopted a five-point rating scale, ranging from “strongly disagree” to “strongly agree”, to evaluate how much participants agreed with the different gratification statements [7]. Questions were amended to specifically concentrate on WeChat use and the students’ psychological needs. The questions were finally passed a pilot test and were translated directly from English into Chinese so as to ensure the reliability and validity.

Exploratory factor analysis with a varimax rotation finally created six distinct constructs, accounting for 78.08 percent of the variance (see Table 2). The motivation of social interaction, characterized by conversations with friends and family members, contains both affection and recognition needs. The most significant uses of WeChat tend to be related to the gratifications for recognition needs (eigenvalue = 11.02, Cronbach’s alpha = 0.90, variance explained: 14.08 percent). Specifically, students use the social media site principally to develop self-confidence, win respect and support, build their own personal identity gain and so on (M = 2.96, SD = 0.89). Affection needs (eigenvalue = 1.56, Cronbach’s alpha = 0.90, variance explained: 12.51 percent) encompass four statements focusing on the capability of the WeChat to help users to give and receive a certain amount of interacting with people about whom they cared (M = 3.30, SD = 0.87). Meanwhile, two requirements of recreational gratifications, which have a clear focus on entertainment and fashion/status in the research. Fashion/status (eigenvalue = 1.90, Cronbach’s alpha = 0.94, variance explained: 13.81 percent) is related to look cool, fashion and stylish (M = 2.68, SD = 0.90). Entertainment (eigenvalue = 0.81, Cronbach’s alpha = 0.76, variance explained: 8.77 percent) comprises three items (e.g. ‘pass time’ and ‘have fun’) (M = 3.40, SD = 0.82). Technological convenience accessibility (eigenvalue = 1.05, Cronbach’s alpha = 0.85, variance explained: 10.90 percent), includes four items that estimate how the WeChat assists users respond to friends and strangers anytime and anywhere (M = 3.38, SD = 0.83). Finally, gratifications for information exchange as the last factor, cognition needs (eigenvalue = 0.85, Cronbach’s alpha = 0.88, variance explained: 10.42 percent), include four items underscoring the helpfulness of the new media technology for enlarging the knowledge range and improving the understanding of the world. Similarity, a composite subscale is created on the basis of the sum of the averaged four items, which demonstrates high internal consistency (M = 3.26, SD = 0.92).

In sum, these four broad classifications of motivations are consistent with the gratifications-sought from the use of social media adopted by prior studies [7, 10, 24, 45]. Moreover, to users, need for technological convenience is reflected to be a special characteristic of motivation in WeChat adoption on mobile phones. Students may seek gratifications through the process of utilizing the new technology to take advantage of the sense of convenience. In addition, recreation and socialization needs are also seen as essential aspects of student’s habitual use of WeChat on mobile devices.

### WeChat use, online political discussion and political efficacy

In predicting the online political discussion and political efficacy, a hierarchical regression was separately run for three political variables: online political discussion, external political efficacy, and internal political efficacy and the results were summed up in Table 3. The integration of predictors significantly predicts political discussion online (R^2^ = 0.70, p < 0.05), but not internal efficacy or external efficacy. Hypotheses H1a, H1b, and H1c hypothesized that intensity of WeChat use would predict online discussion, internal efficacy, as well as external efficacy, respectively. Findings indicate that the association is significant for individual’s online political discussion (b = 0.17, p < 0.05), but not individual’s internal efficacy (b = -0.06, p = 0.86) or external efficacy (b = -0.02, p = 0.49). Thus, H1a is supported, H1b and H1c are not.

**Table 3:** Predictors of online political discussion and political efficacy.

Predictors	Online political discussion		External efficacy		Internal efficacy	
	b	t value	b	t value	b	t value
**Block 1**						
Gender	-0.12	-1.99	0.075	1.17	0.13	1.99
Age	-0.14	-2.08*	-0.23	-3.32*	-0.16	-2.26*
Education	-0.02	-0.36	-0.10	-1.50	0.09	1.23
Household income	-0.01	-0.16	-0.09	-1.42	-0.05	-0.69
Adjusted R^2^	0.04		0.06		0.07	
**Block 2**						
Political interest	0.41	6.47***	0.19	2.95**	0.22	3.28**
Adjusted R^2^	0.20		0.09		0.11	
**Block 3**						
**Social interaction**						
Recognition needs	-0.09	-1.30	.083	1.23	0.158	2.22*
Affection	-0.04	-0.69	-0.10	-1.55	0.019	0.28
**Recreation**						
Entertainments	-0.09	-1.40	-0.06	-0.94	-0.10	-1.44
Fashion or status	0.16	2.65**	0.31	5.09 ***	-0.02	-0.26
**Technological convenience**						
Accessibility	-0.05	-0.74	0.21	3.18**	0.12	1.72
**Information exchange**						
Cognition needs	-0.09	-1.48	0.02	0.24	0.19	2.71**
Adjusted R^2^	0.23		0.22		0.18	
**Block 4**						
Intensity WeChat use	0.17	2.05*	-0.06	-0.69	-0.02	-0.17
Adjusted R^2^	0.23		0.00		0.00	
Total adjusted R^2^	0.70		0.37		0.36	

*p < 0.05

**p < 0.01

***p < 0.001

Results in Table 3 implies the intensity of WeChat usage on mobile phones (β = 0.17, p < 0.05) is the foremost block, explaining 23 percent of the variance. The intensity of WeChat usage is a significant predictor of the level of discussion about government and politics. Motivation is ranked as the second most significant part in explaining 22 percent of the variance in total. However, contrary to what was originally assumed, only fashion/status (β = 0.16, p < 0.01) is a significant predictor of online political discussion. In Block 2, political interest (β = 0.41, p < 0.001), is the third significant predictor of the level of online political discussion. This demonstrates that the more interest in political affairs, the more frequently they participate in online political discussion. H2a and H2b proposed that information requirements will positively link with online political discussion about government and politics, while recreational motives will bring down the linkage. Obviously, findings from the present study do not agree with both assumptions. Finally, demographic is entered into the equation as the fourth most key block contributing a significant proportion of 4 percent. Only age (β = -0.14, p < 0.05) is significant predictors of online political discussion.

In terms of external political efficacy, results show that fashion/ status (β = 0.31, p < 0.001) and accessibility needs (β = 0.21, p < 0.01), as two dimensions of motivations, are significant predictors of the level of external political efficacy. In particular, fashion/ status needs hold strong predictive power at the level of external political efficacy and contribute the majority of the variance. This indicates that the more motivations for fashion and technological convenience in WeChat use, and the higher the level of external political efficacy people has. Besides, age maintains the same predictive role on external political efficacy (β = -0.23, p < 0.05), while interest in politics as the significant predictor (β = 0.19, p < 0.001). For students, recognition need (β = 0.16, p < 0.05) and cognition need (β = 0.18, p < 0.01) are significantly connected to their internal political efficacy. This means that gratifications for seeking information through mobile technology may improve the level of individuals’ internal efficacy. Demographically, age (β = -0.16, p < 0.05) and political interest (β = 0.22, p < 0.01) are significant predictors, indicating that the younger the WeChat users are and the higher political interest they have, the more active they would be in online political activities. H4a is supported, while H4b is not. As a result, of the eight hypotheses proposed in the research, only two show significant.

## Discussion

With the high-speed development of communication networks and mobile phone technology in contemporary China, WeChat has been one of the most eye-catching platforms of interpersonal communication within these few years. The study not only provides empirical evidence of citizen engagement through WeChat in an authoritarian country, but also extends the theory of uses and gratification to the new media adoption in the political life of people. As Campbell & Kwak stated that, the advent of mobile phone technology assures further important step with study and theory on the intersectional field between new media technologies and political participation [49].

This current study systematically investigates how the motivations, political interest, and WeChat use on mobile devices associate with political discussion and political efficacy. Firstly, as expected, the recognition needs including establishing personal identity, gaining respect, and building their confidence are the strongest factors driving Chinese citizens to use WeChat, by contrast with others such as fashion/status, cognition needs, affection, and information-oriented needs. Past studies have also discovered that the purpose of recognition accounted for the most variance in the factor analysis of all those motivation items [45]. Besides, other patterns of psychological motivations were stressed in the former literature on the Internet and mobile phone use studies [7, 49]. For instance, in a study of mobile phone use and gender difference, immediate access and mobility acted as the principal motivations [50]. This result suggests that some newly needs such as accessibility and fashion, invented by the use of new media technologies, could well provide the support and complementarities for the basic needs in people’s everyday life [7]. One explanation of this may be attributed to the truth that modern telecommunication technology constantly offers an ever widening variety of affordances through its different functions and applications to meet young people’s various needs.

Secondly, the study provides first-hand information on the current situation of social networking sites in a non-Western background. Interestingly, fashion needs are the significant predictors of individual’s political discussion online, which embody that keeping up with the recent social trend with their peers facilitated young people’ attention and involvement in the contemporary political discussion. Herein, using the mobile app communication technology for fashion/status is not only a type of entertainment gratifications, but also displays the need for social connection [7]. This conclusion resonates with previous research, which identified that fashion is a form of communication which covers messages of status and power [51]. Moreover, the intensity of WeChat usage is positively related to increase in online political discussion with others. One possible reason is that WeChat is one of the few desirable venues where Chinese citizens’ political views and comments can be expressed and exchanged more freely. Therefore, this finding demonstrates the key role of mobile device in boost people’s civic life in an developing country like China [4].

Thirdly, the research discovers that accessibility needs (the motivations for technological convenience) and fashion needs (one motivation for pleasure-seeking) have the fundamental role in making connections between people and external political efficacy. Fashion-oriented motives promote an increase in the level of external efficacy and consciousness about current public problems among students. Technological convenience fosters interpersonal interaction and mutual support, which can reinforce bonds within the community and improve one’s sense of the government [7]. However, those two motivations are not linked to internal political efficacy. Internal political efficacy is significantly predicted by two additional motivations, including recognition needs and cognition needs. That is, the more WeChat users’ recognition needs are gratified; the more they are likely to acquire confidence that they could comprehend the current political realities in anytime and anywhere. Although Chinese citizens who are stimulated to pursue important political messages may become more informed about the current politics, and thus having more confidence that getting clear on politics, the quite opposite appears to be case for those who apply the new communication tools for more trivial or amusement purposes [24].

This present study makes significant contributions to the growing research in mobile app communications in terms of revealing how specific mobile app technologies affect political discussion and political efficacy among Chinese young citizens. Moreover, it is plausible that those who become more active in using their mobile app tend to be involved in mass-mediated and interpersonal political communication online. New emerging mobile app, especially WeChat seems to fill the void and satisfy the different needs of millions of Chinese young citizens who aspire to push the development of the political democratization in China. A great many scholars and researchers have undertaken lots of discussions and studies on the association between government, media, and society in the context of China at the macroscopic level [15]. From the micro-level, this study offers a fresh insight to the analysis of the influence of WeChat use and subsequent outcomes in the uses and gratifications of theoretical framework. Furthermore, conclusions in the study demonstrate that different requirement types, can bring about radical results, which might not be evident if scholars only gauged frequently of media technology use [24].

## Limitations and suggestions for future research

As an exploratory study, some limitations and suggestions for the future work must be recognized. Firstly, the study concentrates on college students due to time and financial constraints. Therefore, it should be noted that the findings cannot be generalized to the entire population of young people in China. Not only the use of the new communication technology may differ between students and young people who are employed [52], but also political discussion and efficacy are issues related to all young people, attitudes and activities in different age groups ought to be involved in future research and evaluation. Moreover, the study successfully draws on the idea of the Facebook Use Intensity Scale under the background of the intercultural in China, but neglects the influence of different patterns of media contact on political variables. Thus, it is necessary for follow-up studies to solve whether the intensity of media use scale has multidimensional instead of a single one-dimensional test, and seek the corresponding countermeasures. Because that the use of multiple items respectively to gauge the most critical variables will also improve the reliability of the results [24]. Finally, this study emphasizes only how the intensity of WeChat usage would link to political variables based on cross-sectional data. Therefore, exploration on whether social media use behaviors among students over time may increase their involvement in civic and political life is of both practical and theoretical significance. Future empirical studies can build on the empirical evidence of this present study and examine the causal model among these variables in subsequent longitudinal research.
